# Superior Electronic Structure in Two-Dimensional MnPSe_**3**_**/**MoS_2_ van der Waals Heterostructures

**DOI:** 10.1038/s41598-017-10145-z

**Published:** 2017-08-25

**Authors:** Qi Pei, Yan Song, Xiaocha Wang, Jijun Zou, Wenbo Mi

**Affiliations:** 1 Tianjin Key Laboratory of Low Dimensional Materials Physics and Preparation Technology, School of Science, Tianjin University, Tianjin, 300354 China; 2 School of Electrical and Electronic Engineering, Tianjin University of Technology, Tianjin, 300384 China; 3 Key Laboratory for Green Chemical Technology of the Ministry of Education, School of Chemical Engineering and Technology, Tianjin University, Tianjin, 300354 China

## Abstract

We explore the electronic structure of two-dimensional (2D) MnPSe_3_/MoS_2_ van der Waals (vdW) heterostructures based on density functional theory. A novel spin splitting at the valance band maximum of MnPSe_3_ appears in some specific stacking models due to Mn *d* orbital hybridization. The simultaneous spin and valley splitting can be achieved by interfacial coupling, which is attractive for manipulation of the valley and spin degrees of freedom. More importantly, due to the antiferromagnetic ordering of manganese, the opposite spin moments at *K* and *K′* valleys can be observed by transforming configurations, which realizes the tunable spin splitting states. Our theoretical work opens up the opportunities of valley and spin related applications of MnPSe_3_/MoS_2_ vdW heterostructures and offers a practical avenue for exploring novel devices based on the spin and valley degrees of freedom.

## Introduction

Over the last decades, a research upsurge on two-dimensional (2D) materials has emerged due to their remarkable properties and enormous potentials in scalable device applications^1–4^. As a milestone work, the successful stripping of graphene provides a new experimental and theoretical support for the expansion of 2D van der Waals (vdW) material family. Hereafter, a series of graphene-like materials have been fabricated, such as silicene^5^, germanane^6, 7^, phosphorene^8, 9^ hexagonal boron nitride (h-BN)^10, 11^, graphitic carbon nitride (g-C_3_N_4_)^12^ and transition metal dichalcogenides (TMDCs)^13–16^. These 2D materials with different elements exhibit versatile physical and chemical properties^5–16^. However, the magnetism is still a missing property in the current lineup 2D materials. Although the edge structure modification^17^, carrier doping^18^ and transition-metal adsorption^19^ can induce a weak magnetic characteristic, the difficulties in the precise control still hinder the artificial fabrication of these 2D materials. Recently, a newly intrinsic magnetic 2D MoN_2_ has been reported to reveal great potentials in the nanoscale mechanical, electronic and spintronic applications^20^. Consequently, the exploration of 2D spontaneous magnetic crystals is of great importance. In this regard, a new type single-layered magnetic chalcogenophosphates material (exemplified by 2D MnPSe_3_) has been proposed by Li *et al*.^21^, and then a series of 2D *M*P*X*_3_ (*M* = Fe, Mn, Ni, Cd, Zn, *X* = S, Se) have been investigated by Du *et al*.^22^.

Bulk MnPSe_3_ is a layered compound belonging to *M*P*X*_3_ family with a weak interlayer vdW interaction^23^, which makes it feasible to exfoliate few layers and even monolayer experimentally. Similar to TMDCs, 2D MnPSe_3_ crystal also has a hexagonal honeycomb lattice. This kind of Néel antiferromagnetic (AFM) semiconductor shows a direct band gap at its high symmetry points *K* and *K*′^21^. More importantly, the absence of an inversion center and the destruction of time-reversal symmetry are integrated in such a novel system. In previous study^24^, noncollinear calculations performed on monolayer MnPSe_3_ lead to an energy difference between *K* and *K*′ valleys after the bands renormalization, which indicates that MnPSe_3_ is a potential candidate to realize spontaneous valley polarization. However, the Bloch states of two spins at *K* and *K*′ are degenerate in this process. Hence, we propose once the spin splitting can be achieved, the available degree of electron freedoms will be greatly increased.

The strategy of stacking 2D vdW materials with diverse characteristics into their layered heterostructures is widely used in the acquisition of new electronic properties at interface^25^. For instance, He *et al*. have successfully synthesized the ultrathin *p*-GaTe/*n*-MoS_2_ vdw heterostructures with high photovoltaic and photodetecting properties^26^. Chang *et al*. have proposed and verified a light-induced spin Hall effect for interlayer exciton gas in monolayer MoSe_2_/WSe_2_ vdW heterostructures^27^. In our previous work^28^, a tunable spin splitting via perpendicular electric field appearing in arsenene/FeCl_2_ vdW heterostructure has been predicted theoretically. In short, the vdW heterostructures demonstrate some outstanding features beyond its individual components, which can play a vital role in the extension of the electronic degrees of freedom and the development of well-performed electronic devices.

In this work, we comprehensively investigate the electronic structure of 2D MnPSe_3_/MoS_2_ vdW heterostructures with different stacking patterns by density functional theory. In addition, we propose a strategy to realize simultaneously the spin and valley degeneracy splitting in monolayer MnPSe_3_ by interfacial coupling interaction. A spin splitting appears at the valance band maximum (VBM) of MnPSe_3_ in some particular stacking patterns due to the hybridization of Mn *d* orbital, which enriches the available degree of electron freedom. Particularly, the opposite spin moments at *K* and *K′* valleys can be achieved by modulating configurations. The strategies and results illustrated here aimed at a better understanding on the basic properties of MnPSe_3_/MoS_2_ vdW heterostructures and developing the novel spintronic and valleytronic devices. Moreover, our theoretical findings also indicate the MnPSe_3_/MoS_2_ vdW heterostructures could be the potential photocatalyst.

## Calculation details

All the simulations are performed by using Vienna *ab initio* simulation package code^29, 30^ with the generalized gradient approximation (GGA) parameterized by Perdew-Burke-Ernzerhof (PBE)^31^ together with the vdW-D2 correction^32^. The GGA + D2 is used to add the longer-ranged correlation in evaluating vdW interaction between the monolayers. Kohn-Sham single-particle wavefunctions are expanded in the plane wave basis set with a kinetic energy truncation at 500 eV. A 7 × 7 × 1 *k*-point grid centered at Γ point is adopted. The energy and force convergence criteria on each atom are less than 10^−6^ eV and 0.01 eV/Å, respectively. It is well known that GGA cannot properly describe the strongly correlated systems with partially filled *d* subshells. Thus, we use Hubbard *U* terms (5 eV for Mn) to describe the on-site electron-electron Coulomb repulsion as suggested in the literature^33^. The optimized in-plane lattice constants for monolayer MnPSe_3_ and MoS_2_ are 6.403 and 3.189 Å, respectively, which are highly consistent with previously calculated results^21, 34^. The electronic band structures of isolated MnPSe_3_ and MoS_2_ are calculated with and without spin-orbit coupling (SOC) correction. Since SOC has great effects on the band dispersion, it will be used in the simulation progress for heterostrucutres. MnPSe_3_/MoS_2_ vdW heterostructures are constructed based on supercell models, where 2 × 2 × 1 MoS_2_ are stacked on the top of 1 × 1 × 1 MnPSe_3_ unit cell to match with each other. The lattice mismatch for supercells is only 0.4%. We choose an average lattice constant of 6.390 Å as the starting point of the optimization for heterostructures. In order to minimize the interaction between periodic images, a 20-Å vacuum slab is inserted perpendicularly. Meanwhile, the corrections of vdW-D2, Coulomb repulsion *U* and SOC are conducted simultaneously through the whole calculation process so as to guarantee the meaningful comparison of energies.

## Results and Discussion

**Figure 1:**
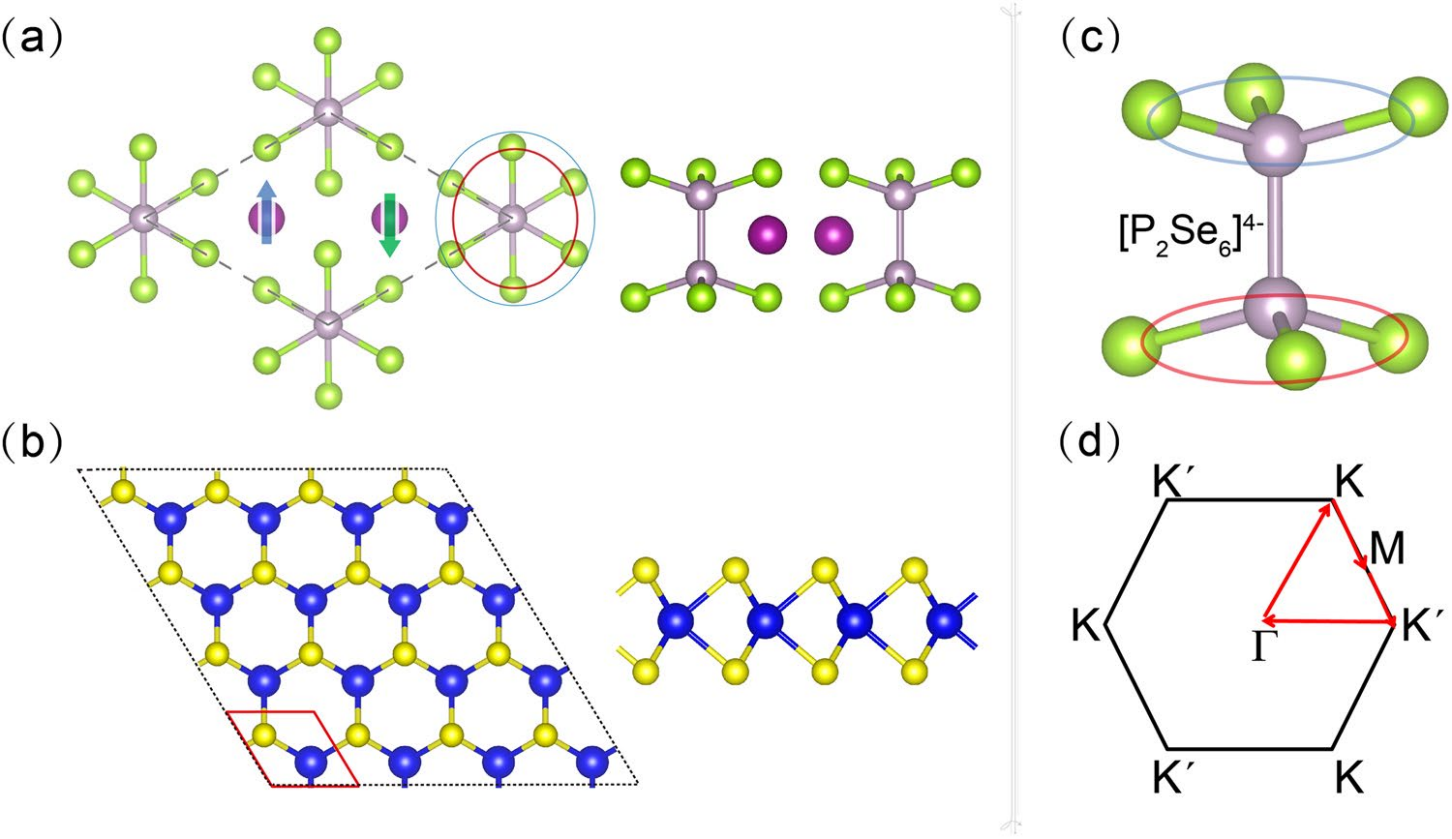
Top and side view of monolayer (**a**) MnPSe_3_ and (**b**) MoS_2_. Blue up-arrow and green down-arrow denote the antiferromagnetic order in MnPSe_3_. The red parallelogram indicates a MoS_2_ unit cell. The lattice structure of [P_2_Se_6_]^4-^ unit in MnPSe_3_ is given in (**c**). First Brillouin zone and high-symmetry points of honeycomb lattice are illustrated in (**d**).

Bulk MnPSe_3_ is an antiferromagnetic semiconductor with a Néel temperature of 74 K^35^. This layered crystal shows a weak interlayer interaction, so that the monolayer MnPSe_3_ can be obtained through exfoliation method. Our calculation results show that the AFM state is more stable than the FM state by about 40 meV per unit cell for 2D MnPSe_3_, indicating the bulk antiferromagnetic characteristic is preserved in monolayer structure. In Fig. 1(a), each unit cell in the monolayer MnPSe_3_ is composed of two Mn^2+^ ions and one [P_2_Se_6_]^4-^ cluster (partial magnification shown in Fig. 1(c)). Each Mn^2+^ ion, assuming an *S* = 5/2 high-spin state, is antiferromagnetically coupled to the three neighboring Mn^2+^ ions, forming an AFM ordering. In addition, the spin density mainly localizes at two Mn ions with the opposite spin magnetic moments, while P and Se atoms have the negligible magnetic moments. The calculated Mn magnetic moment of 4.6 μ_B_ per ion is consistent with previous calculation values of 4.6 μ_B_^24^ and 4.54 μ_B_^36^. These results reveal that the parameters adopted in the present work are valid and reliable.

Bulk MoS_2_ has the 2 *H* stacking order with an inversion symmetric space group ${D}_{6h}^{4}$. When it is exfoliated into monolayer, the lattice symmetry will reduce to ${D}_{3h}^{1}$, accompanied by an definitely broken of inversion symmetry. The honeycomb crystal structure of 2D MoS_2_ is clearly illustrated by the top and side views in Fig. 1(b), where Mo is bonded to six neighboring S atoms. Moreover, the first Brillouin zone and high-symmetry points of honeycomb lattice crystal are described in Fig. 1(d).

**Figure 2:**
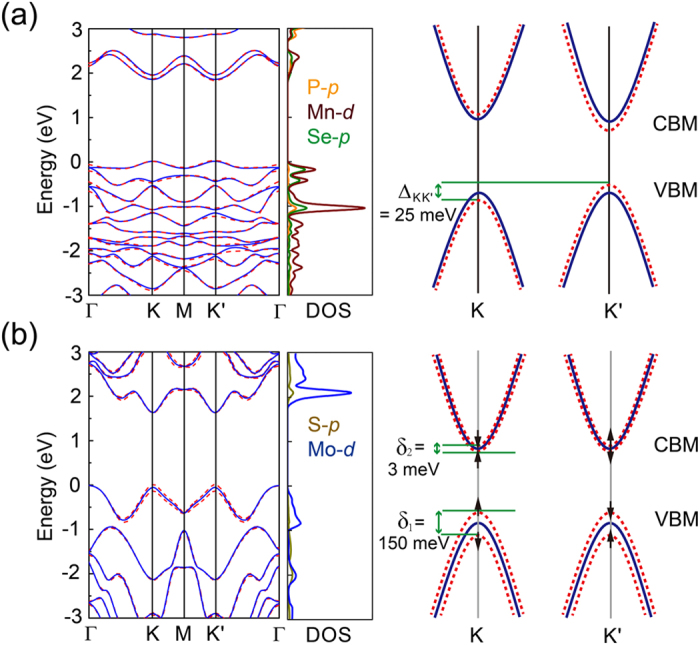
Electronic band structure calculated with (red dashed line) and without (blue solid line) SOC and partial DOS calculated with SOC of monolayer (**a**) MnPSe_3_ and (**b**) MoS_2_. Corresponding up and down opening parabolas are used to represent the minimum conduction and maximum valence bands at two valleys. ↑ and ↓ assume spin direction. Δ_KK′_, δ_1_ and δ_2_ denote valley splitting and spin splitting at *K* and *K′*, respectively.

We lay the main emphasis on the influences of SOC towards the MnPSe_3_ band structures, which is considered to have limited effects thus often be neglected in the previous calculations^21, 37^. Band structures with (red dashed line) and without (blue solid line) SOC correction of the 2D MnPSe_3_ are illustrated in Fig. 2(a). The MnPSe_3_ shows a direct band gap of 1.84 eV without SOC, which falls well within the optical absorption range, facilitating the optical polarization of charge carriers. Our band gap result agrees with the result calculated with PBE functional in the literature^24^, but expectedly smaller than band gaps calculated with the screened hybrid HSE06 functional (2.62 eV in ref. 21 and 2.32 eV in ref. 37), since the PBE functional always underestimates the band gap due to self-interaction errors^38–40^. Beyond that, we can also observe the two spins in the momentum space *K* and *K*′ are both degenerated no matter with or without SOC. Especially, bands of MnPSe_3_ become renormalized by considering SOC and the band gap reduces to 1.80 eV, resulting in an energy difference of about 25 meV between *K* and *K*′ valleys (represented by ${{\rm{\Delta }}}_{KK\text{'}}$). This difference can meet the demand of lifting valley degeneracy for the utilization of the valley degree of freedom. Moreover, from the calculated atoms partial density of states (DOS), it can be concluded that VBM of monolayer MnPSe_3_ are mainly contributed by Se *p* and Mn *d* orbitals, whereas the conduction band minimum (CBM) consists of P *p*, Se *p* and Mn *d* orbitals.

Figure 2(b) presents the band changes of monolayer MoS_2_ (1 × 1 unit cell) with or without SOC. The band structure of monolayer MoS_2_ exhibits a direct band gap of 1.58 eV when SOC is considered, where its VBM and CBM are both located at *K* point, as shown by our PBE calculations. Owing to the strong SOC originated from Mo *d* orbital^41^, the lack of inversion symmetry along with strong SOC leads to the change of spin splitting (*δ*_1_ = 150 meV and *δ*_2_ = 3 meV for valance bands (VB) and conduction bands (CB), respectively) from *K* to *K′*. Meantime, the spin moments of two valleys are opposite. Such phenomenon is consistent with earlier study^42^. Since the SOC interactions have great influences on describing the electronic properties of monolayer MnPSe_3_ and MoS_2_ accurately, SOC corrections are taken into account in all the models through the whole simulations.

**Figure 3:**
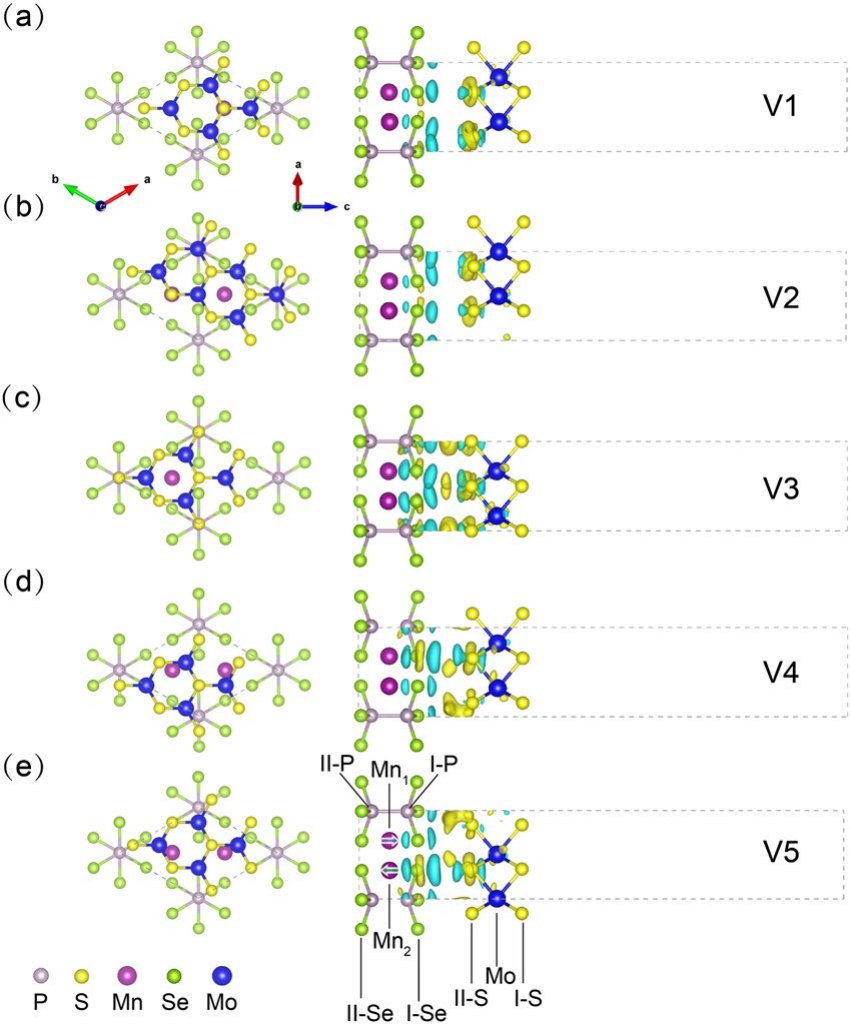
Structure and side view of the charge density difference of MnPSe_3_/MoS_2_ heterostructures with different stacking models V1-V5. The isosurface value is 0.15 e/nm^3^. Yellow (blue) regions represent the net charge gain (loss).

According to the different atoms (Mn, P and Se) right below Mo and S, 2D MnPSe_3_/MoS_2_ heterostructures can be divided into five different stacking models, named as V1-V5. In Fig. 3, each model is interconvertible by horizontal layer sliding. After fully relaxing the structure, the interfacial binding energies (${E}_{{\rm{b}}}$) are calculated by the equation of ${E}_{b}={E}_{MnPS{e}_{3}/Mo{S}_{2}}-({E}_{MnPS{e}_{3}}+{E}_{Mo{S}_{2}})$, where ${E}_{MnPS{e}_{3}/Mo{S}_{2}}$, ${E}_{MnPS{e}_{3}}$ and ${E}_{Mo{S}_{2}}$ represent the total energy of heterostructures, isolated MnPSe_3_ and MoS_2_, respectively. The binding energies of five models (V1-V5) are −447, −426, −411, −408 and −418 meV, respectively, reflecting the weak vdW interaction between the MoS_2_ layer and MnPSe_3_ layer. According to the order from the most stable model to the least stable one, we have the sequence of V4 < V3 < V5 < V2 < V1. It should be noted that the binding energies of V1-V5 are very close, which have the same order in magnitude (10^2^ meV) as other vdW heterostrucutres^43, 44^. Apart from that, the equilibrium interlayer distances are in the range from 3.395 to 3.527 Å. These results evidently prove that 2D MnPSe_3_/MoS_2_ vdW interfaces mainly interact by weak vdW forces.

In order to better illustrate the binding mechanism, the charge density difference of V1-V5 models are calculated by ${\rm{\Delta }}\rho ={\rho }_{MnPS{e}_{3}/Mo{S}_{2}}-{\rho }_{MnPS{e}_{3}}-{\rho }_{Mo{S}_{2}}$, where ${\rho }_{MnPS{e}_{3}/Mo{S}_{2}}$, ${\rho }_{MnPS{e}_{3}}$ and ${\rho }_{Mo{S}_{2}}$ represent the charge densities of heterostructures, isolated MnPSe_3_ and MoS_2_, respectively. The calculated results are shown in Fig. 3. Clearly, the charge redistribution situations can be separated into two main categories, one is related to V1 and V2, whose charge accumulations appear around II-S, Mo and I-Se atoms while charge depletes in the region above and below the S and Se atoms, The other includes V3, V4 and V5, whose aggregation and dissipation of charge are mainly concentrated between the interfacial layer atoms (II-S~I-Se). However, since the isosurface value is only 0.15 e/nm^3^, the charge redistribution is actually insignificant. For the purpose of quantification, the bader charge analysis^45–47^ is also calculated for V1-V5 systems. We take V1 as an example, where hardly any magnetic moment (0.001 μ_B_) is induced in MoS_2_, and the charge transfer (0.01 e) between MnPSe_3_ and MoS_2_ can also be neglected. Since the absence of covalent bonding upon the hybrid interface, the interaction between MnPSe_3_ and MoS_2_ layers is relatively weak.

**Figure 4:**
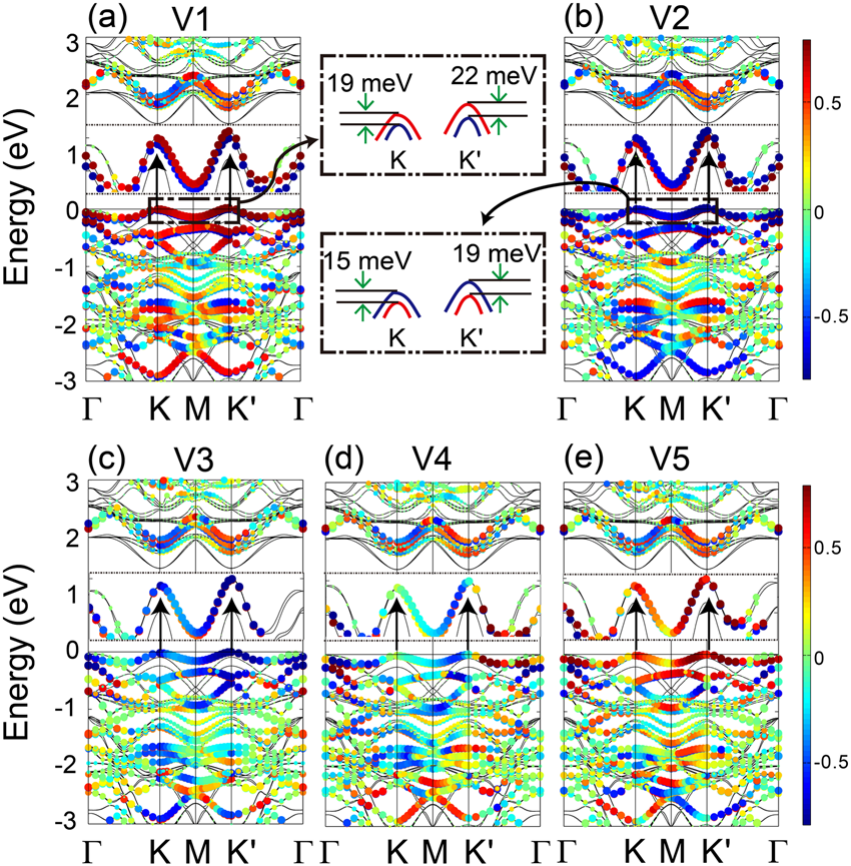
Band structure of V1-V5 models. The circles represent the MnPSe_3_ component and the spin projection along z direction is depicted as the color scale. The size of circles is proportional to orbital components. The insertions in each graph magnify the bands near Fermi level. Corresponding down opening parabolas are schematic drawings of the valance band edge structures at two valleys. Red and blue parabolas represent spin-up and spin-down, respectively.

In order to clearly demonstrate the interaction between MnPSe_3_ and MoS_2_, it is insightful to analyze their electronic structures. Band structures of V1-V5 models are shown in Fig. 4(a)–(e), respectively. Spin projection along *z* direction is depicted as the color scales. The size of circles is proportional to orbital components and the color transition from red to blue represent a transitional form between spin-up and spin-down. We mainly focus on the band alignment near Fermi level. Compared with isolated monolayer MoS_2_, the bands of MoS_2_ component in all the considered heterostructures move down by 0.2 eV relative to Fermi level. Synchronously, the semiconducting nature and the valley features (${{\rm{\Delta }}}_{KK\text{'}}$) of MnPSe_3_ are preserved in the five stacking patterns. As mentioned above, the spin states at *K* and *K′* points are degenerated for isolated MnPSe_3_, while the spin degeneracy of the bands is lifted in V1 and V2 models, leading to a noticeable spin splitting at VBM. In V1 model (Fig. 4(a)), the splitting energy at *K* and *K′* points are calculated as 19 and 22 meV, respectively. Similar to V1 model, this feature of spin splitting is also observed in V2 model (Fig. 4(b)), where the splitting energy is 15 meV at *K* point and 19 meV at *K′* point. It is worth noting that the spin moments are completely opposite at VBM for the two different stacking models. We also give the band structures of the other three models for comparison, while no obvious spin splitting can be observed near Fermi level.

**Figure 5:**
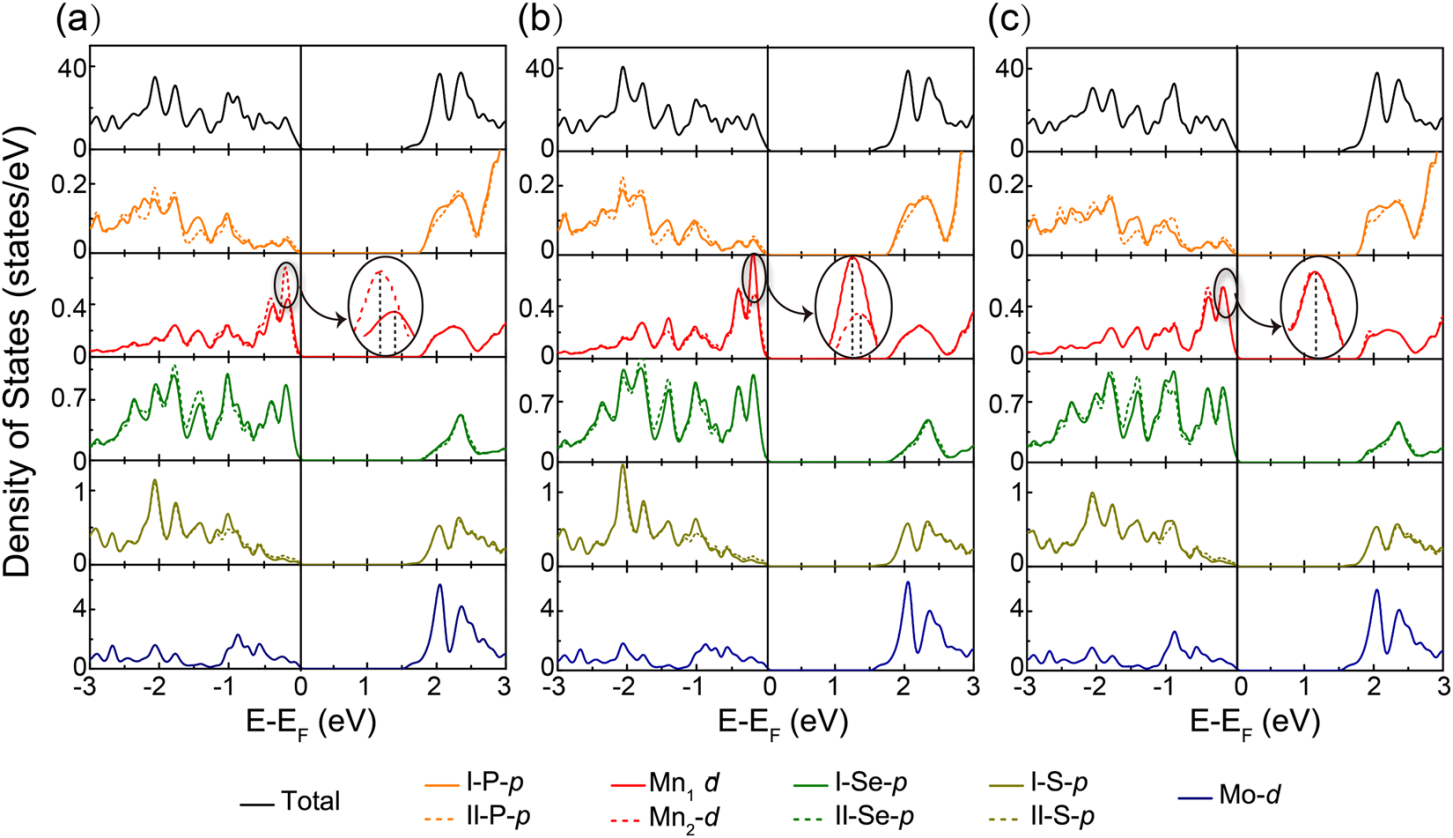
Total and partial DOS of (**a**) V1, (**b**) V2 and (**c**) V4 configurations. Fermi level is indicated by the vertical shadow-line and set to zero.

The origins of these unique properties are further investigated by calculating total DOS and partial DOS of each atom in all models. Owing to the three main kinds of phenomena in the band structures, we choose V1, V2 and V4 models as objects of the study, as displayed in Fig. 5. It can be clearly observed that the curves of Mn_1_-*d* and Mn_2_-*d* states show a peak movement in the range of −0.2~0 eV in the insertions of Fig. 5(a) and (b). As a result, the peak movements of Mn_1_-*d* and Mn_2_-*d* near Fermi level leads to spin splitting at VBM, which can be attributed to the Mn *d* states hybridizing with MoS_2_. However, for other three stacking patterns (represented by V4), the corresponding peak positions are still overlapping like the isolated MnPSe_3_ thus the spin splitting is hardly detected. What′s more, the opposite spin moments of V1 and V2 can be ascribed into the opposite peak position of Mn_1_ *d* and Mn_2_ *d* states in the two models, which can be well exhibited by the partial magnification in the black oval in Fig. 5. Since the two Mn atoms in MnPSe_3_ are antiferromagnetic coupling, the densities for two spins (up and down) on Mn are indeed well separated and localized on two Mn^2+^ ions. Therefore, the peak of Mn_1_ *d* orbital is closer to Fermi level, leading to spin-up states at VBM in V1 model. On the contrary, V2 shows spin-down states at VBM because the peak of Mn_2_ *d* orbital is closer to Fermi level.

In previous theoretical developments^24^, we have had insights into the degree of freedom characterized by the spin and valley indices in 2D MnPSe_3_, where both valleys can absorb the circular polarized light. However, in our calculated system, the spin degeneracy of MnPSe_3_ disappears due to the hybridization of Mn *d* orbital after contacting with MoS_2_, leading to the different excitation energies for spin-up and spin-down electrons at *K* and *K′* valleys. Thus, the excitation of one spin at one valley can be realized selectively in combination with the polarization and frequency of the incident light, which may produce a spin-polarized Hall current. Meanwhile, the simultaneous spin and valley polarization can facilitate the manipulation of spin and valley degrees of freedom by electrical gating in addition to optical pumping. In such a system, some intriguing phenomena, for example, the anomalous valley/spin Hall effect could occur.

**Figure 6:**
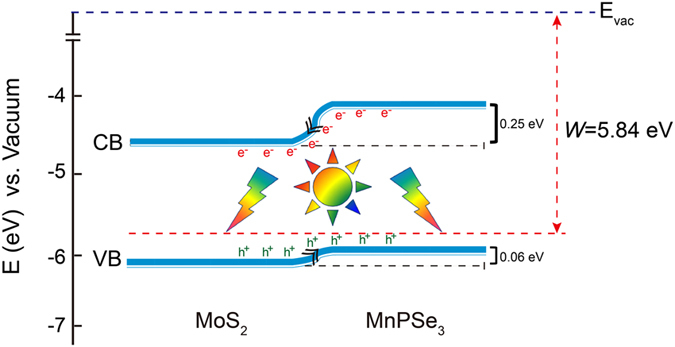
Schematic illustration of band alignment and carrier transfer in MnPSe_3_/MoS_2_ heterostructures.

Among the discussions above mentioned, one point that the MnPSe_3_/MoS_2_ composite is a type-II heterostructure should be worthy of note. We calculate the work functions to achieve the band edges relative to the vacuum potential. The schematic illustration of band alignment and carrier transfer in MnPSe_3_/MoS_2_ heterostructures is shown in Fig. 6. When MnPSe_3_ and MoS_2_ are brought into contact, the Fermi level should be at equilibrium conditions, leading to the CB and VB of MnPSe_3_ more positive than the corresponding bands of MoS_2_. The top part of the VB in heterostructures is mainly contributed by MnPSe_3_, while the states from the MoS_2_ are located at a relatively low position (0.06 eV). On the other hand, the bottom part of the CB mainly comes from the MoS_2_ states while the position of MnPSe_3_ is 0.25 eV higher than MoS_2_. Consequently, the electrons photoexcited from the CB of MnPSe_3_ can be easily transferred into the CB of MoS_2_, leaving the holes in the VB. In other words, this predicted type-II band alignment ensures the photogenerated electrons can easily migrate from MnPSe_3_ into MoS_2_, which can effectively promote the separation of photoinduced carriers. To date, MoS_2_ has received a lot of experimental attention as a cocatalyst in heterostructures^48, 49^. Therefore, our predictions not only provide a mechanistic insight to the possibility that MnPSe_3_/MoS_2_ heterostructures can be a potential photocatalyst, but also can be a guide to extend for future studies of other layered heterostructures in experiment.

## Conclusion

In summary, we propose a strategy to realize the spin and valley degeneracy splitting simultaneously by the interfacial coupling in MnPSe_3_/MoS_2_ heterostructures. The main properties and electronic structures of isolate monolayer MnPSe_3_ and MoS_2_ are preserved in heterostructures due to the weak vdW interaction. In particular, we can obtain the completely opposite spin splitting appearing at VBM by modulating the relative stacking position between MnPSe_3_ and MoS_2_. For V1 model, the splitting energy at *K* and *K′* valley are calculated as 19 and 22 meV, respectively, which are 15 and 19 meV for V2 model. Moreover, the predicted type II band alignment in this hybrid layered heterostructure is of great benefit to the light absorbance and electrons injection, which could be applied in the photoelectrochemical field. These theoretical predictions provide insight to understand the basic properties of 2D MnPSe_3_/MoS_2_ vdW heterostructures and are attractive for manipulation of the valley and spin degrees of freedom.
